# Efficacy of biologics for alveolar ridge preservation/reconstruction and implant site development: An American Academy of Periodontology best evidence systematic review

**DOI:** 10.1002/JPER.22-0069

**Published:** 2022-10-24

**Authors:** Fernando Suárez‐López del Amo, Alberto Monje

**Affiliations:** ^1^ Private practice Madrid Spain; ^2^ Department of Periodontology International University of Catalonia (UIC) Barcelona Spain; ^3^ Department of Periodontics and Oral Medicine University of Michigan Ann Arbor Michigan USA; ^4^ Division of Periodontology CICOM‐MONJE Institute Badajoz Spain; ^5^ Department of Periodontics University of Bern Bern Switzerland

**Keywords:** alveolar ridge augmentation, dental implants, jaw, edentulous, sinus floor augmentation

## Abstract

**Background:**

The use of biologics may be indicated for alveolar ridge preservation (ARP) and reconstruction (ARR), and implant site development (ISD). The present systematic review aimed to analyze the effect of autologous blood‐derived products (ABPs), enamel matrix derivative (EMD), recombinant human platelet‐derived growth factor‐BB (rhPDGF‐BB), and recombinant human bone morphogenetic protein‐2 (rhBMP‐2), on the outcomes of ARP/ARR and ISD therapy (i.e., alveolar ridge augmentation [ARA] and maxillary sinus floor augmentation [MSFA]).

**Methods:**

An electronic search for eligible articles published from January 2000 to October 2021 was conducted. Randomized clinical trials evaluating the efficacy of ABPs, EMD, rhBMP‐2, and rhPDGF‐BB for ARP/ARR and ISD were included according to pre‐established eligibility criteria. Data on linear and volumetric dimensional changes, histomorphometric findings, and a variety of secondary outcomes (i.e., clinical, implant‐related, digital imaging, safety, and patient‐reported outcome measures [PROMs]) were extracted and critically analyzed. Risk of bias assessment of the selected investigations was also conducted.

**Results:**

A total of 39 articles were included and analyzed qualitatively. Due to the high level of heterogeneity across studies, quantitative analyses were not feasible. Most studies in the topic of ARP/ARR revealed that the use of biologics rendered similar results compared with conventional protocols. However, when juxtaposed to unassisted healing or socket filling using collagen sponges, the application of biologics did contribute to attenuate post‐extraction alveolar ridge atrophy in most investigations. Additionally, histomorphometric outcomes were positively influenced by the application of biologics. The use of biologics in ARA interventions did not yield superior clinical or radiographic outcomes compared with control therapies. Nevertheless, ABPs enhanced new bone formation and reduced the likelihood of early wound dehiscence. The use of biologics in MSFA interventions did not translate into superior clinical or radiographic outcomes. It was observed, though, that the use of some biologics may promote bone formation during earlier stages of healing. Only four clinical investigations evaluated PROMs and reported a modest beneficial impact of the use of biologics on pain and swelling. No severe adverse events in association with the use of the biologics evaluated in this systematic review were noted.

**Conclusions:**

Outcomes of therapy after post‐extraction ARP/ARR and ARA in edentulous ridges were comparable among different therapeutic modalities evaluated in this systematic review. Nevertheless, the use of biologics (i.e., PRF, EMD, rhPDGF‐BB, and rhBMP‐2) in combination with a bone graft material generally results into superior histomorphometric outcomes and faster wound healing compared with control groups.

## 1 INTRODUCTION

Decades of investigation have demonstrated that dental implants are a predictable and effective therapy for the rehabilitation of partially and completely edentulous patients.^1^, ^2^ However, insufficient or inadequate bone volume derived from pathological processes (e.g., chronic disease progression), congenital conditions, undesirable events (e.g., trauma), or therapeutic interventions (e.g., tooth extraction or resective surgical procedures) often represents a common challenge in clinical practice. The presence of limited bone volume may interfere with ideal positioning of the implant and, subsequently, compromise the ability to achieve and maintain optimal long‐term peri‐implant health, function, and esthetics. Alveolar ridge preservation (ARP) or reconstruction (ARR) and implant site development (ISD) techniques are used to correct and overcome these limitations. Under the umbrella of ARP/ARR and ISD there are a variety of procedures and techniques that share a common objective, the provision of a recipient site that is adequate for implant placement in the ideal position. More specifically, ARP aims at attenuating post‐extraction dimensional changes in intact or mostly intact sites, while ARR is indicated in extraction sites presenting extensive alveolar bone damage. On the other hand, ISD aims at the correction of hard and soft tissue deficiencies in healed, edentulous alveolar ridges.

Regarding hard tissue, horizontal and vertical alveolar ridge augmentation (ARA), as well as maxillary sinus floor augmentation (MSFA) arguably represent the core ISD interventions in contemporary clinical practice. These interventions, along with ARP/ARR, can be performed with a variety of techniques and materials, each presenting specific distinctions and limitations. Absorbable and non‐absorbable barrier membranes, particulate bone replacement graft materials with different origins, and autologous bone blocks are among the most frequently employed materials for bone augmentation in ISD and ARP. While proven successful in multitude of investigations,^3^, ^4^, ^5^, ^6^ all bone preservation and augmentation protocols present with drawbacks and limitations, potentially including, but not limited to, complications during the healing phase (e.g., infection), reduced amount of new bone formation, and delayed healing. The use of biologics has been proposed with the purpose of overcoming these limitations and increase the predictability of therapy.

Biologics are a group of agents or mediators that exert a biological effect through various mechanisms to promote tissue regeneration. Biologics promote a variety of essential cellular events in wound healing including deoxyribonucleic acid (DNA) synthesis, chemotaxis, cell differentiation, mitogenesis, and matrix biosynthesis.^7^, ^8^ Consequently, these biologics have been used to enhance the outcomes of bone regeneration procedures.^9^, ^10^ Also, biologics have attributed to a variety of additional beneficial properties such as reduced local inflammation and reduced postoperative pain, among others.^11^, ^12^

The use of biologics in periodontics and implant dentistry has been extensively studied. Nevertheless, there is still controversy regarding their true potential and clinical indications. Consequently, in alignment with the purpose of the American Academy of Periodontology (AAP) Best Evidence Consensus (BEC) on the use of biologics in contemporary clinical practice, the aim of this systematic review was to investigate the effect of commonly used biologics (i.e., autologous blood‐derived products [ABPs], enamel matrix derivative [EMD], recombinant human platelet‐derived growth factor‐BB [rhPDGF‐BB], and recombinant human bone morphogenetic protein‐2 [rhBMP‐2]) on the outcomes of different ARP/ARR and ISD modalities (i.e., ARA, and MSFA) by addressing the following focused question: Does the use of ABPs, EMD, rhPDGF‐BB, or rhBMP‐2, either as a monotherapy or in combination with scaffolds or graft materials, render superior outcomes after the performance of ARP/ARR and ISD procedures compared with a control group with standard treatment protocols not involving the use of biologics?

## 2 MATERIALS AND METHODS

The protocol of this study was designed in accordance with the Cochrane Handbook for Systematic Reviews of Interventions^13^ and the Preferred Reporting Items for Systematic Reviews and Meta‐Analysis (PRISMA) 2020 guidelines.^14^

### 2.1 Population, Intervention, Comparison, Outcome (PICO) question

- Population: Adult individuals
- Intervention: Use of ABPs, EMD, rhBMP‐2, or rhPDGF‐BB in ARP/ARR, ARA, or MSFA.
- Comparison: Conventional ARP/ARR and ISD modalities not involving the use of biologic mediators. All three treatments (ARP/ARR, ARA, and MSFA) were evaluated individually.
- Outcomes:
  1. Primary: Bone changes (dimensional changes compared with baseline records [linear and/or volumetric measurements obtained before the grafting procedure] and histomorphometric data).
  2. Secondary: Clinical, implant‐related, digital imaging, safety, and patient‐reported outcome measures (PROMs). Clinical outcomes involved structural and biological assessments performed during direct or indirect clinical examination. Digital imaging refers to the assessment of bone and soft tissue via radiographs, digital imaging, and communications in medicine (DICOM) and/or stereolithography (STL) files. Histologic evaluation involved the use of qualitative (descriptive histology) and/or quantitative measurements (e.g., histomorphometric). PROMs are assessments performed by the patients.

### 2.2 Eligibility criteria

Human randomized clinical trials (RCTs) with parallel‐arm or split‐mouth design published in the English language after January 1, 2000 were screened. Eligibility criteria were: (1) surgical treatment of adult patients (≥18 years of age) presenting single or multiple extraction sites or edentulous areas in need of implant‐supported/‐retained rehabilitation; (2) minimum of 10 sites per study arm; (3) minimum follow‐up of 2 months for ARP/ARR; (4) minimum follow‐up of 4 months for MSFA and ARA; (5) one study arm involved the use of a biologic (i.e., ABPs, EMD, rhBMP‐2, or rhPDGF‐BB), either as a monotherapy or combined with other modalities of treatment while another arm consisted of conventional therapy without the use of biologics; and (6) report at least one of the following outcomes of interest: dimensional bone changes or histomorphometric data.

### 2.3 Information sources

An electronic literature search was conducted independently by two authors (F.S.L.A. and A.M.) in several databases including MEDLINE (via PubMed), EMBASE, and Cochrane Central Register of Controlled Trials (CENTRAL) to identify eligible articles published up to November 1, 2021. Bibliographies of the identified articles as well as previously published systematic reviews in these topics were also searched.^15^, ^16^, ^17^, ^18^, ^19^, ^20^

### 2.4 Article selection process

Two independent reviewers (F.S.L.A. and A.M.) performed the hand search and read the title and abstract of the entries obtained from the literature search. After completing the screening, both reviewers assessed the full‐text version of potentially eligible studies for final article selection. Disagreements were resolved by open discussion. If no consensus could be reached, an independent referee (Gustavo Avila‐Ortiz) was consulted. Any missing information that could contribute to this systematic review was requested from the corresponding author(s) via email communication.

### 2.5 Electronic literature search strategy

The PubMed search strategy was: (((((((((((((((((((((((((((((edentulous jaw[MeSH Terms]) OR (edentulous mouth[MeSH Terms])) AND (edentulous alveolar ridge)) OR (alveolar ridge augmentation[MeSH Terms])) OR (mandibular ridge augmentation[MeSH Terms])) OR (maxillary ridge augmentation[MeSH Terms])) OR (maxillary sinus floor augmentation[MeSH Terms])) OR (sinus floor augmentation[MeSH Terms])) OR (sinus floor elevation[Title])) OR (alveolar ridge preservation[Title])) OR (socket preservation[Title])) OR (horizontal ridge augmentation[Title])) OR (horizontal bone augmentation[Title])) OR (vertical ridge augmentation[Title])) AND (vertical bone augmentation[Title])) OR (platelet growth factor[Title/Abstract])) OR (enamel matrix derivative[Title/Abstract])) OR (platelet derived growth factor[Title/Abstract])) OR (EMD[Title/Abstract])) OR (Emdogain[Title/Abstract])) OR (PDGF[Title/Abstract])) OR (PRP[Title/Abstract])) OR (PPP[Title/Abstract])) OR (PRF[Title/Abstract])) OR (platelet rich fibrin[Title/Abstract])) OR (GEM‐21[Title/Abstract])) OR (bone morphogenetic protein [Title/Abstract])) AND (bone augmentation[Title/Abstract])) OR (bone gain[Title/Abstract])) OR (implant survival[Title/Abstract])) OR (bone loss[Title/Abstract]). Note that combinations of MeSH and EMTREE terms and keywords were prioritized. Moreover, a less specific screening using non‐MeSH index terms was conducted to expand the search scope. This included the “type of intervention” AND “a biologic” (e.g., ridge augmentation AND platelet‐derived growth factor). A similar strategy was used in EMBASE and Cochrane library using the filter for randomized clinical trials.

### 2.6 Data extraction

The following data were extracted and recorded in duplicate by two independent reviewers (F.S.L.A. and A.M.): (1) citation, and year of publication; (2) study location: country and type of setting (e.g., private practice, university, military, or dental hospital); (3) type of procedure and approach; (4) characteristics of participants (i.e., sample size [initial and final number of participants per arm], sex and age distribution per arm); (5) characteristics of interventions: test and control groups; (6) outcome measures of interest; and (7) source of funding.

### 2.7 Methodological quality and risk of bias assessment

The assessment of methodological quality and risk of bias of each included RCT was performed in duplicate using the Cochrane risk‐of‐bias tool for randomized trials (RoB1)^21^ which provided guidelines for the following parameters: (1) random sequence generation; (2) allocation concealment method; (3) blinding of participants and personnel; (4) blinding of outcome assessment; (5) incomplete outcome data; (6) selective reporting; and (7) other bias.

### 2.8 Data synthesis

Data were collated into evidence tables and presented according to the objective/indication of the surgical intervention of interest. The descriptive analysis was structured by type of ISD procedure and divided into the following categories: study characteristics, population characteristics, intervention characteristics, and effect of biologic on treatment outcomes.

In addition, based on the criteria established by the adapted version^22^ of the American Dental Association (ADA) Clinical Practice Guidelines Handbook (see Tables S1–S3 in online *Journal of Periodontology*),^23^ critical assessment of the literature and strength of recommendation were applied to the extracted data and results presented in this systematic review. These recommendations were presented according to the following set of criteria:

- **Clinical comparisons and main findings**: Description of the comparisons (i.e., therapies involving the use of biologics vs. controls) and outcomes of interest, based on the main findings of individual studies and pooled estimates (if available). This description was structured as described above: by type of intervention divided into four different categories.
- **Level of certainty**: Assessment of the extent to which there is confidence in the estimate of the effect of therapy considering the best available evidence. Briefly, this assessment is dictated by the following domains: (a) risk of methodological bias; (b) applicability of evidence; (c) inconsistency or unexplained heterogeneity of results; (d) imprecision (wide confidence intervals); and (e) high probability of publication bias (e.g., selective reporting). Level of certainty may be classified as: high, moderate, or low (see Tables S1 and S2 online *Journal of Periodontology*).
- **Net benefit rating (benefit‐harm estimation)**: Whether the expected benefits outweigh the potential for harm.
- **Adverse events and complications**: Relevant adverse events and complications.
- **Strength of clinical recommendation**: This assessment reflects the extent to which one can be confident that adherence to the treatment recommendation will be more beneficial than harmful, considering the strengths and weaknesses of the best available evidence. Strength of clinical recommendation may be classified as: strong, in favor, weak, expert opinion for/supports, expert opinion questions the use, expert opinion against, or against (see Table S3 in online *Journal of Periodontology*).

## 3 RESULTS

**FIGURE 1:**
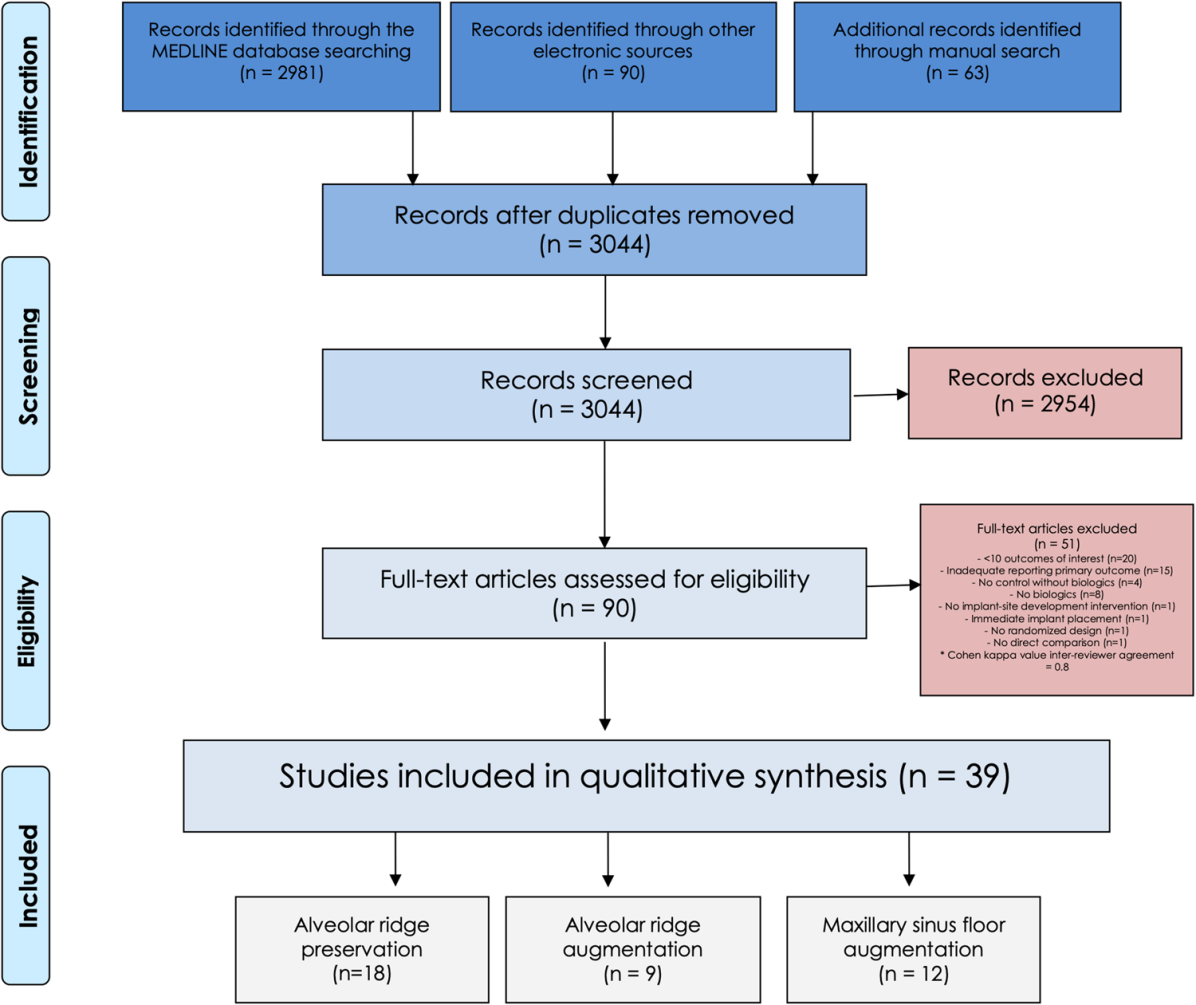
PRISMA flowchart

The PRISMA flowchart for literature selection is depicted in Figure 1. In summary, 3044 records were identified after removal of duplicates. Among them, 90 were assessed for full‐text and 39 were included in the qualitative synthesis (18 in ARP/ARR, nine in ARA, and 12 in MSFA). A summary with the characteristics of the included investigations is presented in Tables 1, 2, and 3. The most frequent reason for exclusion based on full‐text evaluation was insufficient sample size (*n* = 20) followed by inadequate report of the primary outcome (*n* = 15). The complete list of excluded articles is displayed in Table S4 in the online *Journal of Periodontology*.

**TABLE 1:** Characteristics of included articles on the topic of alveolar ridge preservation

Authors	1. Setting(s) 2. Country(ies)	RCT Design	Protocol for socket orifice closure	Biologic	Interventions & Material	Final Number of Participants	Final number of sockets	Healing Time (months)	Method(s) for assessment of primary outcomes	Main findings
Alzahrani et al. (2017)	1. University 2. Saudi Arabia	Parallel arms	None, sutures	PRF	None	12	12	2	Cast	PRF was associated with less bone resorption compared to UH.
				None	UH	12	12			
Areewong et al. (2019)	1. University 2. Thailand	Parallel arms	None, sutures	PRF	None	18	15	2	Biopsy	Higher new bone formation ratio with PRF without statistically significant difference compared to UH.
				None	UH	15	13			
Badakhshan et al. (2020)	1. Private practice 2. Iran	Split‐mouth	None, sutures	L‐PRF	None	19	22	3	Cast (via 3D analysis)	L‐PRF significantly reduced bone resorption compared to UH.
				None	UH		22			
Canellas et al. (2020)	1. University 2. Brazil	Parallel arms	None, sutures	L‐PRF	None	23	23	3	Biopsy & CBCT	Statistically significant higher new bone formation with L‐PRF compared to UH. L‐PRF significantly reduced bone resorption compared to UH.
				None	UH	22	22			
Castro et al. (2020)	1. University 2. Belgium	Split‐mouth	None, sutures	L‐PRF	None	20	20	3	CBCT	PRF failed to attenuate dimensional changes. Similar bone resorption for L‐PRF and A‐PRF+ compared to UH. No statistically significant differences found amongst groups.
				A‐PRF+	None		20			
				None	UH		20			
Clark et al. (2018)	1. University 2. USA	Parallel arms	Collaplug & Cyanoacrylate	A‐PRF	None	10	10	3‐4	Biopsy & Clinical	A‐PRF and A‐PRF + FDBA significantly reduced the loss of ridge height compared to UH. Similar outcomes for A‐PRF and FDBA. A‐PRF demonstrated the highest percentage of new bone formation.
				A‐PRF	FDBA	10	10			
				None	FDBA	10	10			
				None	UH	10	10			
Coomes et al. 2014	1. University 2. USA	Parallel arms	None, sutures	1.5 mg/mL rhBMP‐2	ACS	20 (18 clinical)	20 (18 clinical)	5	CBCT & clinical	Clinical and radiographic analyses demonstrated superior outcomes (less resorption and greater reconstruction of buccal wall) for the rhBMP‐2 group. Multiple of these comparisons reached statistically significant differences between groups. 12% of patients in the test group reported mild adverse event (erythema and localized swelling) versus 0% in control group.
				None	CS	18 (16 clinical)	18 (16 clinical)			
Fiorellini et al. 2005	1. Multicenter 2. USA	Parallel arms	Primary wound closure	1.5 mg/ml rhBMP‐2	ACS	21	95	4	CT	Groups involving the use of rhBMP‐2 demonstrated greater bone formation and less bone resorption. Particularly, the use of 1.50 mg/ml rhBMP‐2 exhibited more favorable outcomes reaching statistically significant differences in multitude of analysis when compared to the other groups, including the sockets treated with 0.75 mg/ml rhBMP‐2. Two hundred and fifty adverse events were reported for 78 subjects. The most common were edema, pain, and erythema. The groups involving the use of rhBMP‐2 reported a greater number of cases with edema and erythema.
				0.75 mg/ml rhBMP‐2	ACS	22				
				none	ACS	17				
				None	None	20				
Huh et al. 2011	1. Multicenter 2. South Korea	Parallel arms	None, sutures	1.5 mg/ml rhBMP‐2	β‐TCP + HA	36	36	3	CT	Statistically significant superior results for the test group regarding changes in bone width and height. No adverse events to the grafted material observed.
				None	β‐TCP + HA	36	36			
Kim et al. 2014	1. Multicenter 2. South Korea	Parallel arms	Collagen membrane + primary wound closure	0.05 mg/mL rhBMP‐2	DBM	29	29	3	CT	rhBMP‐2 group exhibited marginally less dimensional collapse compared to DBM alone. No statistically significant differences observed between test and control groups regarding vertical and horizontal bone resorption. No adverse events reported.
				None	DBM	30	30			
Kumar et al. (2018)	1. University 2. India	Parallel arms	None, sutures	PRF	None	48	30	6	Clinical & Questionaries	PRF and PRF + CSH yielded similar outcomes compared to UH. PRF groups reported more favorable outcomes regarding postoperative pain compared to UH.
				PRF	CSH		30			
				None	UH		30			
Lee et al. (2020)	1. University 2. South Korea	Parallel arms	Collagen membrane	EMD	DBBM	15	15	5	CBCT & Questionnaires	Similar dimensional changes for EMD + DBBM compared to DBBM. Duration of postoperative pain and swelling significantly reduced with the use of EMD.
				None	DBBM	15	15			
Lin et al. (2021)	1. University 2. China	Parallel arms	CGFs, sutures	CGFs	DBBM	12	12	8	Biopsy	New bone formation was significantly greater for CGFs/DBBM compared to DBBM. Significantly less percentage of residual graft in CGFs/DBBM compared to DBBM.
			Collagen membrane	None	DBBM	12	12			
			None	None	UH	12	12			
Mercado et al. (2021)	1. Private practice 2. Australia	Parallel arms	Free mucosal graft	EMD	DBBM	21	21	4	Biopsy & CBCT	Similar dimensional changes for EMD + DBBM compared to DBBM. Significantly more new bone formation, less residual graft, and less non‐mineralized tissue and marrow spaces for EMD + DBBM compared to DBBM.
				None	DBBM	21	21			
Stumbras et al. (2020)	1. University 2. Lithuania	Parallel arms	Collagen membrane	None	BBM	10	10	3	Biopsy	The PRGF group demonstrated the highest percentage of newly formed bone and the lowest percentage of non‐mineralized tissue reaching statistical significance. The control group also demonstrated significantly more new bone formation compared to BBM and FDBA groups.
				None	FDBA	10	10			
			None, sutures	PRGF	None	10	10			
				None	UH	10	10			
Stumbras et al. (2021)	1. University 2. Lithuania	Parallel arms	Collagen membrane	None	BBM	10	10	3	CBCT	PRGF yielded similar results compared to BBM and FDBA. Both PRGF and BBM groups demonstrated statistically significant less reduction in ridge width compared to UH.
				None	FDBA	10	10			
			None, sutures	PRGF	None	10	10			
				None	UH	10	10			
Suttapreyasri and Leepong (2013)	1. University 2. Thailand	Split‐mouth	None, sutures	PRF	None	8	10	2	Cast	PRF demonstrated similar outcomes than UH.
				None	UH		10			
Temmerman et al. (2016)	1. University 2. Belgium	Split‐mouth	None, sutures	L‐PRF	None	22	22	3	CBCT & Questionnaires	L‐PRF was associated with statistically significant less ridge width changes compared to UH. L‐PRF reduced postoperative pain compared to UH.
				None	UH		22			

PRF, platelet‐rich fibrin; UH, unassisted healing; L‐PRF, leucocyte platelet‐rich fibrin; CBCT, cone beam computed tomography; A‐PRF+, advanced platelet‐rich fibrin+; A‐PRF, advanced platelet‐rich fibrin; FDBA, freeze‐dried bone allograft; CSH, calcium sulphate hemihydrate; EMD, enamel matrix derivative; DBBM, deproteinized bovine bone mineral; CGFs, concentrated growth factors; BBM, bovine bone mineral; PRGF, plasma rich in growth factors; rhBMP‐2, human bone morphogenetic protein 2; CT, computed tomography; ACS: absorbable collagen sponge; ß‐TCP, ß‐tricalcium phosphate; HA, hydroxyapatite; DBM, demineralized bone matrix; CS, collagen sponge.

**TABLE 2:** Characteristics of included articles on the topic of alveolar ridge augmentation

Authors	1. Setting(s) 2. Country(ies)	RCT Design	Intervention	Biologic	Material/carrier	Final Number of Participants	Final number of interventions	Healing Time (months)	Method(s) for assessment of primary outcomes	Main findings from primary outcomes
Eskan et al. (2014)	1. University 2. United States	Parallel arms	HRA: Ridge augmentation by means of PLC‐membrane	PRP	ALL	16	16	4	Caliper and histology	PRP enhances bone formation and results in increased horizontal bone gain and percentage vital bone.
				None	ALL	16	16			
Hartlev et al. (2019)	1. University 2. Denmark	Parallel arms	HRA: Autogenous bone block graft covered by either a PRF membrane (test group) or an DBBM and a resorbable collagen barrier membrane (control group)	PRF	AB block	27	27	6	CBCT	PRF does not add any further benefit in terms of of bone gain
				None	AB block	27	27			
de Freitas et al. (2013)	1. University 2. Brasil	Parallel‐arms	HRA: Ti‐Mesh and rhBMP‐2/ACS (1.5mg/ml) or titanium mesh and autogenous bone harvested from the retromolar area	BMP‐2	ACS	12	12	6	CBCT	BMP‐2 does not provide significant benefits in terms of bone gain
				None	AB (mandibular ramus)	12	12	6		
Isik et al. (2021)	1. University 2. Turkey	Double‐center parallel‐arms	HRA: Ridge augmentation simultaneous to implant placement. No barrier membrane	PRF	DBBM	20	50	6	CBCT	Liquid PRF does not contribute to bone gain
				None	DBBM	20	48			
Jung et al. (2009)	1. University 2. Switzerland	Split‐mouth	HRA: DBBM + collagen membrane +rhBMP‐2 (0.18mg) and DBBM + collagen membrane (control)	BMP‐2	DBBM	11	11	6	Clinical and radiographic examination	Implants placed in bone augmented with DBBM, a collagen membrane and rhBMP‐ 2 revealed excellent clinical and radiological outcomes after 3 and 5 years, equal to controls.
				None	DBBM		11			
Nam et al. (2017)	1. University 2. South Korea	Parallel‐arms	HRA/VRA: Envelope approach with no vertical releasing incisions and no barrier membrane	BMP‐2 (0.5mg)	HA	10	10	4	CT	The use of BMP‐2 seems to exert a negligible role in the early outcomes at regenerated sites. No major adverse events were linked with the use of BMP‐2
				None	DBBM	10	10	4		
Santana & Santana (2015)	1. University 2. Brazil	Parallel arms	HRA: Autogenous bone block grafts harvested from the mandibular ramus (control) vs. TCP + PDGF and PTFE membrane (test)	PDGF	TCP	15	15	6	Caliper	PDGF combined with TCP may be a suitable alternative for AB block grafts
				None	AB (mandibular ramus)	15	15			
Thoma et al. (2018)	1. University 2. Switzerland	Double‐center parallel‐arms	HRA: DBBM block soaked in BMP‐2 (test) vs. symphysis or retromolar autogenous bone block (control)	BMP‐2 (1.5mg/dL)	DBBM block	12	12	4	Clinical, PROMs, histomorphometric	Similar outcomes were achieved by both groups in terms of bone gain. PROMs slightly favored the test group but histological outcomes indicated that the control group tended to exhibit greater rate of mineralized tissue
				None	AB block	12	12			
Torres et al. (2010)	1. University 2. Spain	Parallel arms	HRA/VRA: DBBM used with a Ti‐Mesh. In the test group PRP placed on the top of the Ti‐Mesh	PRP	DBBM	15	22	6	CBCT	Applying PRP over the Ti mesh, may prevent complications, such as mesh exposure and graft failure.
				None	DBBM	15	21			
				None	DBBM	5	5			
				None	DBBM		22			

Abbreviations: PRF, platelet‐rich fibrin; PRP, platelet‐rich plasma; L‐PRF, leucocyte platelet‐rich fibrin; CBCT, cone beam computed tomography; ALL, allograft; AB, autologous bone; DBBM, deproteinized bovine bone mineral; AB, autologous bone; PRGF, plasma rich in growth factors; LA, lateral‐wall approach; HRA, horizontal ridge augmentation; VRA, vertical ridge augmentation; TCP, tricalcium phosphate; PDGF, platelet‐derived growth factor; PTFE, polytetrafluoroethylene; Ti, titanium.

**TABLE 3:** Characteristics of included articles on the topic of maxillary sinus floor augmentation

Authors	1. Setting(s) 2. Country(ies)	RCT Design	Protocol for sinus floor elevation	Biologic	Material	Final Number of Participants	Final number of interventions	Healing Time (months)	Method(s) for assessment of primary outcomes	Main findings from primary outcomes
Barros Mourão et al. (2019)	1. University 2. Brazil	Split‐mouth	LA: Osteotomy performed with Piezoelectric. Resorbable membrane between the material and the Schneiderian membrane	BDGF	CaP	10	10	6	CBCT	BDGF does not improve bone repair when associated with calcium phosphate in MSFA procedures
				None	CaP		10			
Bettega et al. (2009)	1. Private practice 2. France	Split‐mouth	LA: not specified	PRP	AB (iliac crest)	18	18	12	CT and histology	PRP does not provide any further benefit for bone healing
				None	AB (iliac crest)		18			
Boyne et al. (2005)	1. University 2. USA	Parallel‐group	LA: Osteotomy performed with a bur and the lateral bony wall was removed	BMP‐2 (0.75mg/mL)	ACS	18	18	4	CT and histology	BMP‐2 is safe and effective to induce bone formation in maxillary sinus floor elevation procedures to enable implant placement
				BMP‐2 (1.5mg/dl)	ACS	17	17			
				None	AB and/or ALL	13	13			
Cho et al. (2020)	1. University 2. South Korea	Double‐center parallel‐arms	CA: Special drilling system with hydraulic system to lift‐up the membrane	PRF	None	20	20	12	CBCT	PRF provided superior support for the elevated sinus membrane
				None	None	20	20			
Froum et al. (2013) (A)	1. University 2. USA	Split‐mouth	LA: Rotatory bur or piezoelectric for osteotomy.	PDGF	DBBM	24	24	4‐5 or 7‐9	Histology	More rapid formation of vital bone with the addition of rhPDGF may allow for earlier implant placement
				None	DBBM		24			
Froum et al. (2013)(B)	1. University 2. USA	Split‐mouth	LA: Osteotomy performed with rotary bur or piezoelectric	BMP‐2/ACS (8.4mg and 5.6 mL)	ALL	21	10	6 to 9	Histology	The group with higher dose of rhBMP‐2 combined with ALL had more newly formed bone and less residual ALL particles when compared to the group with the lower dose combined with ALL and to the control group
				BMP‐2/ACS (4.2mg and 2.8 mL)	ALL		11			
				None	ALL		11			
Kim et al. (2015)	1. University 2. South Korea	Multi‐center parallel‐arm	LA: not specified	BMP‐2 (1mg/mL)	HA	65	65	3	Histology	The use of BMP‐2 is safe, effective and accelerates bone formation in the early stages of healing after MSFA
				None	DBBM	62	62			
Nizam et al. (2018)	1. University 2. Turkey	Split‐mouth	LA: Osteotomy performed with rotatory burs. Resorbable membrane adapted to the sinus wall	PRF	DBBM	13	13	6	Histology	RF in ABBM does not improve the amount of regenerated bone or the amount of the graft integrated into the newly formed bone
				None	DBBM		13			
Pichotano et al. (2019)	1. University 2. Brazil	Split‐mouth	LA: Osteotomy performed with rotatory burs. Resorbable membrane adapted to the sinus wall	L‐PRF	DBBM	12	12	4 (test) 8 (control)	CBCT, histology and RFA	The addition of L‐PRF to DBBM for MSFA allows early implant placement with increased new bone formation than ABBM alone after 8 months of healing
				None	DBBM		12			
Triplett et al. (2009)	1. University 2. USA	Multi‐center parallel‐arm	LA: Osteotomy performed with a bur and the lateral bony wall was removed	BMP‐2 (1.5mg/dL)	ACS	58	58	6	CT and histology	BMP‐2 is safe and effective in MSFA with no marked differences in terms of newly bone formation. Vertical bone gain was superior for the control group.
				None	AB	69	69			
Wiltfang et al. (2003)	1. University 2. Germany	Parallel arms	LA: not specified	PRP	TCP	22	22	6	Histology	PRP does not significantly contribute to bone regeneration in MSFA
				None	TCP	23	23			
Zhang et al. (2012)	1. University 2. China	Parallel arms	LA: Osteotomy prepared for access.	PRF	DBBM	5	6	6	Histology	PRF does not contribute to bone regeneration in MSFA
				None	DBBM	5	5			
				None	DBBM		22			

Abbreviations: PRF, platelet‐rich fibrin; PRP, platelet‐rich plasma; L‐PRF, leucocyte platelet‐rich fibrin; CBCT, cone beam computed tomography; FDBA, freeze‐dried bone allograft; CSH, calcium sulfate hemihydrate; EMD, enamel matrix derivative; DBBM, deproteinized bovine bone mineral; BDGF, blood‐derived growth factors; AB, autologous bone; ALL, allogenic bone; ACS, absorbable collagen sponge; PRGF, plasma rich in growth factors; LA, lateral‐wall approach; CA, crestal approach; CaP, calcium phosphate; TCP, tricalcium phosphate; RFA, resonance frequency analysis; MSFA, maxillary sinus floor augmentation.

Due to the significant heterogeneity across articles (e.g., discrepancies between experimental and control groups, diversity of biologics used, and different grafting procedures), a quantitative synthesis of the data reported in the included studies and, consequently, a meta‐analysis could not be completed. Instead, a descriptive but thorough analysis of the reported outcomes was performed. It is important to highlight that certain biologics were used off label in some of the selected studies.

### 3.1 Study characteristics

#### 3.1.1 Alveolar ridge preservation

Year of publication ranged from 2005 to 2021. A total of 18 investigations were included of which 14 were RCTs with a parallel‐arm design,^9^, ^11^, ^24^, ^25^, ^26^, ^27^, ^28^, ^29^, ^30^, ^31^, ^32^, ^33^, ^34^, ^35^ while only four were split‐mouth.^36^, ^37^, ^38^, ^39^ (Table 1) Two studies were performed in a private practice setting^9^, ^36^ 13 were performed in a university setting,^11^, ^24^, ^25^, ^26^, ^27^, ^28^, ^29^, ^30^, ^31^, ^32^, ^37^, ^38^, ^39^ while the remaining were multicenter.^33^, ^34^, ^35^ These studies were conducted in different countries without predominance of one particular location. The most frequent method of assessment was three‐dimensional radiography. Other methods included analysis of biopsies, periapical radiographs, casts (physical or digital), and clinical measurements. Only three investigations evaluated PROMS. ^11^, ^27^, ^39^ Nevertheless, most studies used a combination of the above‐mentioned methods for assessing ISD outcomes. Two studies evaluated the same sample of patients providing histomorphometric^29^ and cone‐beam computed tomography (CBCT)^28^ data separately. The number of sockets evaluated for each particular intervention among the different studies ranged from 10 to 36. Healing time ranged from 2 to 8 months, being 3 to 4 months the most frequently reported healing period in a total of 11 studies. ^9^, ^25^, ^26^, ^28^, ^29^, ^33^, ^34^, ^35^, ^36^, ^37^, ^39^

#### 3.1.2 Alveolar ridge augmentation

Year of publication ranged from 2010 to 2021. Overall, nine studies^10^, ^12^, ^40^, ^41^, ^42^, ^43^, ^44^, ^45^, ^46^ were included and all were designed as parallel‐arm RCTs (Table 2). All the studies were performed in university settings with no predominant geographical location. The most frequent method of assessment was three‐dimensional radiographic methods, including computed tomography and CBCT (*n* = 5).^12^, ^40^, ^41^, ^44^, ^46^ Clinical assessments using a caliper were performed in two studies^10^, ^42^ and only in two studies histomorphometric assessments were performed.^10^, ^43^ PROMs was assessed in one study.^43^

#### 3.1.3 Maxillary sinus floor augmentation

Year of publication ranged from 2003 to 2020. A total of 12 articles were selected of which six^47^, ^48^, ^49^, ^50^, ^51^, ^52^ reported split‐mouth and six^53^, ^54^, ^55^, ^56^, ^57^, ^58^ parallel‐arms studies (Table 3). All the studies, but one that was performed in private practice,^48^ were conducted in university settings. The most frequent method of assessment was histomorphometry of bone biopsies.^48^, ^49^, ^50^, ^51^, ^52^, ^54^, ^55^, ^56^, ^57^, ^58^ The second most prevalent method of assessment was three‐dimensional radiography.^47^, ^48^, ^51^, ^53^, ^56^, ^58^ PROMS were not assessed in any of the selected studies in this category.

### 3.2 Population characteristics

#### 3.2.1 Alveolar ridge preservation

A total of 656 patients providing 807 sockets were evaluated. Only seven studies reported dropouts^11^, ^25^, ^26^, ^28^, ^29^, ^32^, ^33^, ^37^ accounting for 27 patients and 29 sockets failing to be analyzed. It is important to highlight that two different articles by Stumbras and colleagues reported different outcomes of the same sample of patients.^28^, ^29^ Most studies reported a mean age for the subjects evaluated, generally ranging from 40 to 60 years. Only one investigation presented with great discrepancy from the above‐mentioned range, reporting a mean of 22.62 ± 2.44 years.^38^ Similarly, most studies reported a comparable distribution of patients between both sexes. Smokers were included in seven studies,^11^, ^24^, ^28^, ^29^, ^30^, ^32^, ^37^ excluded in six,^9^, ^25^, ^26^, ^31^, ^36^, ^39^ and not reported in five.^27^, ^33^, ^34^, ^35^, ^38^

#### 3.2.2 Alveolar ridge augmentation

In total, 231 patients were evaluated. These contributed to 320 sites. Only four dropouts from one study were noted.^10^ The age ranged from 19 to 76 years. Females contributed slightly higher to the sample when compared with males. Light smokers (≤10 cig</day) were included in one study.^12^

#### 3.2.3 Maxillary sinus floor augmentation

Overall, 323 patients for a total of 502 maxillary sinuses, were evaluated. Only two dropouts from one study were noted.^53^ Males and females contributed equally to the total sample. While two studies^54^, ^55^ did not provide information on the inclusion of smokers, one study^49^ stated that light smokers (≤10 cig</day) were included.

### 3.3 Intervention characteristics

#### 3.3.1 Alveolar ridge preservation

Most of the included investigations (12/18) clearly specified the avoidance of flap elevation during the extraction procedure.^11^, ^24^, ^25^, ^26^, ^27^, ^28^, ^29^, ^31^, ^32^, ^36^, ^37^, ^39^ Similarly, most studies evaluated only single extraction sites^9^, ^11^, ^25^, ^26^, ^28^, ^29^, ^30^, ^31^, ^32^, ^33^, ^35^, ^39^ and excluded molars.^9^, ^11^, ^24^, ^25^, ^26^, ^28^, ^29^, ^33^, ^34^, ^36^, ^37^, ^38^, ^39^ Socket wall integrity was not clearly defined and/or reported in most studies. Nevertheless, marked differences were observed amongst included investigations with eligibility criteria ranging from intact or mostly intact socket walls to ≥50% facial bone loss.^24^, ^34^, ^59^ Eleven investigations had two groups or study arms,^9^, ^11^, ^24^, ^25^, ^31^, ^32^, ^33^, ^35^, ^36^, ^38^, ^39^ of which six compared sockets filled with biologics versus unassisted healing.^24^, ^25^, ^31^, ^36^, ^38^, ^39^ All these studies used ABPs. Two studies compared the combination of EMD + collagenated deproteinized bovine bone mineral (DBBM) versus collagenated DBBM alone.^9^, ^11^ The remaining three studies with two groups used rhBMP‐2 in combination with different materials compared with the sole use of a collagen sponge,^32^ β‐tricalcium phosphate (β‐TCP) + hydroxyapatite (HA),^35^ or demineralized bone matrix (DBM) gel.^33^ Three studies presented with three groups or arms. Castro et al. compared two types of ABPs versus unassisted healing.^37^ Kumar et al. compared the following groups: (1) platelet‐rich fibrin (PRF) versus (2) medical grade calcium sulfate hemihydrate covered with PRF versus (3) unassisted healing. ^27^ On the other hand, Lin et al. compared (1) concentrated growth factors (CGFs) combined with DBBM versus (2) DBBM alone versus (3) unassisted healing.^30^ Last, four investigations had four different groups ^26^, ^28^, ^29^. Two of these investigations represent the same sample of patients divided into the following groups: (1) bovine bone mineral (BBM) versus (2) freeze‐dried bone allograft (FDBA) versus (3) plasma rich in growth factors (PRGF) versus (4) unassisted healing.^28^, ^29^ Clark and colleagues compared advanced platelet‐rich fibrin (A‐PRF) alone versus A‐PRF + FDBA versus FDBA versus unassisted healing.^26^ The remaining investigation with four groups by Fiorellini and colleagues compared two groups with different concentrations of rhBMP‐2 (1.5 and 0.75 mg/ml) plus an absorbable collagen sponge (ACS) with a placebo group (ACS alone), and unassisted healing.^34^

Overall, ABPs were the most investigated biologic (12 studies).^24^, ^25^, ^26^, ^27^, ^28^, ^29^, ^30^, ^31^, ^36^, ^37^, ^38^, ^39^ The ABPs studied in these investigations included: PRF, L‐PRF, A‐PRF, A‐PRF+, PRGF, and CGF. On the other hand, EMD was used in two investigations, always as an adjunct,^9^, ^11^ rhBMP‐2 was used in four studies with dosages ranging from 0.05 to 1.5 mg/ml,^32^, ^33^, ^34^, ^35^ and none of the included articles reported the use of rhPDGF‐BB.

Unassisted healing was included as a control group in 13 investigations,^24^, ^25^, ^26^, ^27^, ^28^, ^29^, ^30^, ^31^, ^34^, ^36^, ^37^, ^38^, ^39^ while two studies compared DBBM alone versus DBMM in combination with EMD,^9^, ^11^ and three studies involving the use of rhBMP‐2 reported the sole use of a collagen sponge, β‐TCP + HA, or DBM gel as control groups.^32^, ^33^, ^35^

Most studies did not attempt to obtain primary closure; nor did they use additional materials for socket sealing other than sutures.^24^, ^25^, ^27^, ^31^, ^36^, ^37^, ^38^, ^39^ Nevertheless, it is important to mention that multiple investigations studying ABPs also used this biologic as a membrane to cover the socket orifice. Other studies involved the use of collagen membranes,^11^, ^28^, ^29^, ^30^, ^33^ a rapidly absorbing collagen sponge in combination with cyanoacrylate,^26^ or a free mucosal graft^9^ to cover the socket for one or more of the included groups.

#### 3.3.2 Alveolar ridge augmentation

Overall, seven studies^10^, ^40^, ^41^, ^42^, ^43^, ^45^, ^46^ explored the effects of biologics on horizontal ridge augmentation (HRA), while two studies^12^, ^44^ evaluated HRA and vertical ridge augmentation (VRA). In three studies,^10^, ^41^, ^45^ conventional guided bone regeneration (GBR) by means of an absorbable barrier membrane was performed in the test and control groups. In one study, GBR was only applied in the test group, while the control group consisted of autogenous block grafts harvested from the mandibular ramus.^42^ Further, one study^40^ tested the effect of ABPs in combination with intraoral autogenous block grafts compared with the same intervention, but grafted simultaneously with anorganic bovine bone mineral and covered with a resorbable barrier membrane. The only study that explored the effectiveness of platelet‐rich plasma (PRP) on HRA and VRA used a titanium‐mesh and anorganic bovine bone mineral.^12^ It is worth noting that one study assessed an envelope approach for regeneration using DBBM and rhBMP‐2, but no barrier membrane.^44^ In terms of implant placement stage, one study^41^ reported the use of the biologic with simultaneous implant placement, while all the other included studies involved delayed implant placement.

#### 3.3.3 Maxillary sinus floor augmentation

Only one study^53^ aimed at testing the effect of biologics on transalveolar sinus floor elevation. In this study, a special drilling system incorporating hydraulic properties to lift‐up the membrane was used. Hence, the intervention for the vast majority of the studies was MSFA via lateral window approach, as described elsewhere,^60^ with the osteotomy performed with either a rotatory bur or a piezoelectric instrument. Concerning the bone replacement graft material, six studies combined the biologic with a bone substitute (4 studies^49^, ^50^, ^51^, ^54^ with anorganic bovine bone mineral and two studies^47^, ^55^ with β‐tricalcium phosphate), one with autogenous bone harvested from the iliac crest,^48^ and in one study the biologic was used per se.^53^ Seven studies tested the effect of ABPs,^47^, ^48^, ^50^, ^51^, ^53^, ^54^, ^55^ in particular PRP,^48^, ^55^ PRF,^50^, ^51^, ^53^, ^54^ and blood‐derived growth factors (BDGF).^47^ Only one study tested rhPDGF‐BB^49^ and none explored the effect of EMD on the outcomes of MSFA. Concerning the use of BMPs in MSFA, in two studies,^56^, ^58^ the carrier used was an ACS, while other two studies used allografts and HA.^52^, ^57^

### 3.4 Effect of biologics on treatment outcomes

#### 3.4.1 Alveolar ridge preservation

Eighteen studies reported dimensional and/or histomorphometric changes occurring after tooth extraction. Fifteen investigations evaluated dimensional changes, six of them through clinical measurements or casts analysis^26^, ^27^, ^31^, ^32^, ^36^, ^38^ and the remaining 10 used three‐dimensional radiography (note that Coomes et al. used both methods).^9^, ^11^, ^25^, ^28^, ^32^, ^33^, ^34^, ^35^, ^37^, ^39^ These investigations studied the dimensional changes at different locations, including, but not limited to, vertical collapse at mesial, distal, mid‐buccal, and mid‐lingual aspects, as well as horizontal (width) changes at different levels from the alveolar crest.

In general, selected investigations failed to demonstrate superior outcomes in association with the use of biologics when compared with conventional approaches.^9^, ^11^, ^26^, ^28^ Nevertheless, biologics (alone and/or in combination with other graft materials) did contribute in most investigations to attenuate the resorption process that typically occurs after tooth extraction as compared with unassisted healing or with the sole use of an ACS.^25^, ^26^, ^27^, ^28^, ^31^, ^32^, ^34^, ^36^, ^39^ It is important to note that the differences between groups, when present, were mostly associated to changes in both ridge height as well as width in the most coronal aspects of the socket.^26^, ^28^, ^39^

Other investigations reported on alternative methods for assessment such as radiographic bone fill^27^, ^31^, ^37^, ^39^ and evaluation of the so‐called “alveolar bone area”.^30^ These alternative analyses typically resulted in more favorable outcomes for the test group.^30^, ^31^, ^37^, ^39^ Two investigations also reported on early soft tissue wound healing^11^ and dimensions of socket orifice,^38^ both demonstrating no differences between groups.

Histomorphometric assessment of bone biopsies was performed in six investigations.^9^, ^24^, ^25^, ^26^, ^29^, ^30^ Overall, the use of biologics (i.e., ABPs and EMD) seems to have a beneficial effect on mineralized tissue formation with all six studies reporting superior percentages for the groups involving the use of biologic mediators as a monotherapy or in combination with graft materials. These comparisons reached statistically significant differences (to at least one other group) in five investigations.^9^, ^25^, ^26^, ^29^, ^30^ On the other hand, these differences seem to be more modest for non‐mineralized tissue and the diminished presence of residual graft material. Nevertheless, biologics contributed to the remodeling of allogenic and xenogenic grafting materials reporting in general a lower percentage of residual graft that occasionally reached statistical significance.^9^, ^26^, ^30^ Notably, the only study reporting histomorphometric assessment with the use of EMD demonstrated statistically significant differences for all three parameters (greater percentage of mineralized tissue, less residual graft, and less soft tissue marrow spaces) favoring the test group.^9^ It is important to mention that the above‐mentioned comparisons were made to a variety of “control” groups that sometimes involved the use of bone replacement grafts. None of the studies included in this review evaluated the histomorphometric outcomes in extraction sockets treated with rhBMP‐2.

Only three investigations assessed PROMS. Kumar et al. reported more favorable outcomes regarding postoperative pain with the use of PRF, although swelling was more prevalent in one of the groups involving the use of this biologic, likely due to the additional use of calcium sulfate in this particular group.^27^ Temmerman et al. also found differences in terms of postoperative pain favoring the use of L‐PRF.^39^ Lee and colleagues failed to observe differences in pain and swelling severity, but demonstrated statistically significant differences favoring the use of EMD for the duration of pain and swelling.^11^ Overall, the use of biologics appears to be associated with more favorable outcomes regarding postoperative pain; however, these differences seem to be minimal and last only for a limited period of time.

Regarding implant‐related outcome measures, two investigations evaluated the feasibility of implant placement after performing ARP/ARR with rhBMP‐2 + ACS versus different control groups. Both studies reported a greater number of implants installed without the need for further augmentation in the groups involving the use of rhBMP‐2.^32^, ^34^

Last, no adverse events derived from the use of ABPs or EMD were reported. On the other hand, two out of four investigations evaluating the effectiveness and safety of rhBMP‐2 reported adverse events.^32^, ^34^ Coomes et al. reported that 12% of patients in the test group (vs. 0% in the control group) experiencing mild erythema and localized swelling that resolved spontaneously 7‒10 days after the procedure.^32^ Fiorellini and colleagues reported a total of 250 adverse events for 78 out of the 80 subjects evaluated in their investigation. These events were mostly associated with the test groups and primarily consisted of transient postoperative oral edema, pain, and erythema.^34^

#### 3.4.2 Alveolar ridge augmentation

In general, the use of biologics included in test groups did not show superior outcomes in terms of clinical, radiographic, or histologic parameters when compared with the control groups. Nevertheless, it must be noted that one study reported a statistically significant difference in terms of mineralized tissue formation and horizontal bone gain after 4 months of healing, favoring the PRP group.^10^ Interestingly, another study demonstrated that covering the titanium‐mesh with PRP in ARA procedures may lead to significantly less incidence of wound dehiscence, which in turn, may lead to reduced postoperative complications and failure of the regenerative intervention.^12^ No adverse events derived from the use of biologics were reported. Importantly, the use of BMPs was proven safe and effective, but their performance was not superior to the control groups. PROMs revealed slightly enhanced outcomes in terms of postoperative pain after the use of BMPs compared with autogenous block grafts.^43^

#### 3.4.3 Maxillary sinus floor augmentation

The benefit of using ABPs in combination with bone substitutes was clearly demonstrated in one study (L‐PRF).^51^ In summary, a statistically significant difference of ≈14% that favored the test group for newly formed bone and ≈10% that favored the control group for residual bone graft was reported. This study concluded that bone healing can be accelerated by means of combining a bone graft with L‐PRF and that this may lead to earlier implant placement after MSFA. Another study showed modest benefits as only a difference of 8% to 10% in terms of mineralized tissue formation could be seen in favor of the test group (PRP).^55^ It is worth noting that the only study that explored the effect of ABPs versus a “true” control group (saline) resulted in superior outcomes by means of vertical bone gain ≈1mm) in favor of the test therapy. The use of rhPDGF‐BB was tested in one study that revealed that mineralized tissue formation was ≈10% higher in the test group after 4 to 5 months of healing.^49^ Nevertheless, at 7 to 9 months the difference was negligible. In consistency with this finding, greater mineralized tissue formation at early healing time points was observed when rhBMP‐2 was used.^52^, ^57^ No adverse events derived from the use of biologics were reported.

### 3.5 Risk of bias assessment

The results of the risk of bias assessment for the included investigations are summarized in Figure S1 in tonline *Journal of Periodontology*. In the ARP/ARR category, 50% of the studies showed high risk of bias ^11^, ^24^, ^26^, ^27^, ^30^, ^31^, ^36^, ^38^, ^39^ while 50% reported some concerns.^9^, ^25^, ^28^, ^29^, ^32^, ^33^, ^34^, ^35^, ^37^ In studies on the topic or ARA, 100% of the studies exhibited some concerns.^10^, ^12^, ^40^, ^41^, ^42^, ^43^, ^44^, ^45^, ^46^ In the group of MSFA investigations, 92% of the studies presented some concerns, ^47^, ^48^, ^49^, ^50^, ^52^, ^53^, ^54^, ^55^, ^56^, ^57^, ^58^ while 8% showed low risk of bias.^51^

### 3.6 Clinical recommendations

Based on the screened evidence and the results described in this manuscript, strength of clinical recommendation according to the American Dental Association (ADA) Clinical Practice Guidelines Handbook was established. These recommendations were grouped by interventions as follows:

- **Alveolar ridge preservation**
  1. Level of certainty: Low for ABPs (i.e., PRF, L‐PRF, A‐PRF, A‐PRF+, PRGF, and CGF), EMD, and rhBMP‐2.
  2. Net benefit rating (benefit‐harm estimation): For all investigated biologics, modest or uncertain additional clinical benefits outweigh potential harms or benefits balanced with potential harms. ABPs alone generally outperform unassisted healing with regard to dimensional changes. However, the use of ABPs, EMD, and rhBMP‐2 generally fails to promote additional clinical benefits compared with alternative and more conventional graft materials. Regarding histomorphometric outcomes, the use of ABPs and EMD is associated with more favorable results.
  3. Adverse events and complications: No severe adverse events and/or complications related to the use of ABPs, EMD or rhBMP‐2 were reported in the selected studies. Nevertheless, mild inflammatory reactions (e.g., erythema, localized swelling) may occur more frequently with the use of rhBMP‐2. Regarding PROMS, the use of ABPs and EMD seem to exert a favorable but marginal effect that last only for a limited period of time.
  4. Strength of clinical recommendation: Expert opinion supports the use of ABPs, EMD, and rhBMP‐2 for ARP/ARR. Evidence is lacking; the level of certainty is low and, consequently, expert opinion guides the recommendation of this intervention.
- **Alveolar ridge augmentation**
  1. Level of certainty: Low for ABPs (i.e., PRP and PRF), rhPDGF‐BB, and rhBMP‐2
  2. Net benefit rating (benefit‐harm estimation): Modest or uncertain additional clinical benefits outweigh potential harms or benefits balanced with potential harms.
  3. Adverse events and complications: No relevant adverse events and/or complications related to the use of ABPs, rhPDGF‐BB, or rhBMP‐2 were reported in the selected studies. PROMS were assessed in one study reporting slight superiority for the test group using rhBMP‐2.
  4. Strength of clinical recommendation: Expert opinion supports the use of ABPs, rhPDGF‐BB, and rhBMP‐2 for ARA. Evidence is lacking; the level of certainty is low and, consequently, expert opinion guides the recommendation of this intervention.
- **Maxillary sinus floor augmentation**
  1. Level of certainty: Low for ABPs (i.e., PRP, PRF, L‐PRF, and BDGF), rhPDGF‐BB, and rhBMP‐2.
  2. Net benefit rating (benefit‐harm estimation): Modest or uncertain additional clinical benefits outweigh potential harms or benefits balanced with potential harms.
  3. Adverse events and complications: No relevant adverse events and/or complications related to the use of ABPs, rhPDGF‐BB, and rhBMP‐2 were reported in the selected studies. PROMS were not assessed in any of the selected studies on the topic of MSFA.
  4. Strength of clinical recommendation: Expert opinion supports the use of ABPs, rhPDGF‐BB, and rhBMP‐2 for MSFA. Evidence is lacking; the level of certainty is low and, consequently, expert opinion guides the recommendation of this intervention.

## 4 DISCUSSION

### 4.1 Main findings

The demand for ARP/ARR and ISD interventions has increased in recent years due to the popularity of dental implant therapy. Nonetheless, research efforts over the last two decades have been focused on increasing predictability through minimally invasive approaches and the use of biologics to promote enhanced outcomes. The present systematic review aimed at exploring the effect of biologics on ARP/ARR and ISD interventions. Interestingly, it was observed that limited and heterogeneous high‐quality evidence exist, which precluded the conduction of a meta‐analysis. In this sense, it is important to emphasize that the use of certain biologics (i.e., EMD and rhPDGF‐BB) for the studied interventions are considered off‐label. This likely contributed to the heterogeneity of the findings, the marked differences amongst studies, the limited number of investigations, and the lack of evidence evaluating certain therapies (e.g., rhPDGF‐BB for ARP or EMD for MSFA). As such, data extracted from the studies selected should be cautiously interpreted. Nevertheless, studies included in this review reported no adverse events derived from the use of biologics with the exception of rhBMP‐2 in ARP/ARR. These adverse events were more frequently observed in the test groups involving the use of this biologic but were never severe and included most commonly localized edema, pain, and erythema. With regard to ARP/ARR, both ABPs and EMD provide satisfactory outcomes when combined with bone replacement graft materials. Also, ABPs alone outperformed unassisted healing in most studies with regard to dimensional changes after tooth extraction. Similarly, the usage of rhBMP‐2 in combination with either a graft material or an ACS was also associated with favorable results that generally outperformed controls groups. The effectiveness of rhBMP‐2 in ARP/ARR seems to be dose‐dependent. Last, superior histomorphometric outcomes are associated with the use of ABPs and EMD in ARP/ARR. For ARA procedures, rhPDGF‐BB, ABPs, and rhBMP‐2 are effective in promoting bone formation. Similarly, ABPs may be beneficial in terms of higher rate of mineralized tissue formation and lower incidence of early postoperative complications. Regarding MSFA, rhPDGF‐BB, ABPs, and rhBMP‐2 are effective in promoting and accelerating bone formation during the early stages of healing compared with control therapies. The above‐mentioned findings are in general terms aligned with those reported in previous systematic reviews.^17^, ^18^, ^61^, ^62^, ^63^

### 4.2 What is the biologic plausibility of these findings?

Biologics are molecular mediators that regulate cellular events in the wound healing process via established mechanisms of action, which include angiogenesis, osteogenesis, cementogenesis, extracellular matrix formation, and chemotaxis, among other biological processes.^8^, ^63^ Biologics are used in clinical settings to increase predictability and enhance the outcomes of therapy. Nevertheless, different biologics have diverse dominant effects and therefore, their use should be tailored according to the clinical scenario and the desired outcomes. For instance, rhPDGF‐BB, a potent mitogenic agent, is naturally released by blood platelets after binding to specific cell surface receptors.^64^ In vitro, rhPDGF has been shown to promote fibroblast, cementoblast, and osteoblast migration and proliferation.^65^ On the other side, the rationale for the use of ABPs is primarily based on the role that platelets have in hemostasis and for being a natural source of growth factors.^66^ Furthermore, it has been demonstrated in‐vitro that PRF elicits an anti‐inflammatory response in macrophages^67^ and suppresses osteoclastogenesis.^68^ EMD contains naturally occurring proteins such as enamelin, amelogenin, and ameloblastin. This biologic has demonstrated to induce the proliferation of mesenchymal stem cells, as well as enhance osteogenic differentiation by stimulating the proliferation of pre‐osteoblasts and differentiation of osteoblast‐like cells and osteoblasts.^69^, ^70^ Last, rhBMP‐2 belongs to a group of molecules, the bone morphogenetic proteins, the largest subfamily of the transforming growth factor‐β superfamily.^71^ To date, 14 bone morphogenetic proteins have been identified, with rhBMP‐2 and ‐7 being the most extensively used and investigated. These proteins are capable of inducing bone formation by guiding the differentiation of mesenchymal cells into bone and bone marrow cells.^72^ Nevertheless, despite their biological properties and other evidence supporting the clinical use of these biologics, in general terms, findings from this systematic review do not strongly support the use of biologics to optimize the outcomes of ISD interventions.

### 4.3 Recommendations for future investigations

Properly designed RCTs aimed at evaluating the clinical, implant‐related, digital imaging, histologic and patient‐related outcomes of ARP/ARR and ISD procedures involving the use of biologics in different clinical scenarios are warranted. To date, the literature is replete with articles reporting the use of biologics, more specifically, ABPs, EMD, rhPDGF‐BB, and rhBMP‐2 Nevertheless, the great majority of these investigations are case control, case series or case reports.^19^ Although these investigations could provide valuable information, the risk of bias, mainly due to the presence of variables unaccounted for, can be very significant. Consequently, in order to establish guidelines and recommendations for the use of biologics in ARP/ARR and ISD procedures, only a high level of clinical evidence was considered in this systematic review. The strict eligibility criteria unequivocally lead to a limited selection of studies, which may have influenced the outcomes of the review. Future clinical studies should involve groups or study arms as methodologically similar as possible with the only difference being the additional use of a biologic. These studies are expected to further contribute to elucidate the true efficacy of these mediators. Also, the evaluation of PROMs should be routinely considered in future investigations.

### 4.4 Limitations

The main limitations of this systematic review are: (1) The marked methodological heterogeneity across selected investigations that prevented the performance of a quantitative analysis. For the same reason, comparisons between biologics were not feasible; (2) The efficacy of some biologics could not be assessed due to the lack of clinical investigations reporting their usage, for example rhPDGF‐BB for ARP/ARR and EMD for MSFA; (3) Although grouped under the umbrella of biologics, these mediators greatly differ between one another and, therefore, a comparative assessment of reported outcomes should be done with caution. Moreover, although often presented as a consolidated category for the purpose of this review, it must be recognized that ABPs represent a heterogenous group of therapeutic agents. The sole variation in centrifugation protocols can affect their composition and potential for regeneration,^73^ and (4) A variety of patient‐ and site‐specific variables can affect the outcomes of therapy. Including only RCTs can contribute to reduce the likelihood of selection bias; however, some critical parameters, such as the thickness (whenever present) of the facial alveolar bone in extraction sites,^9^, ^74^, ^75^ were not evaluated in most included investigations.

## 5 CONCLUSIONS

Current evidence does not support that the use of ABPs, EMD, rhPDGF‐BB, or rhBMP‐2, either as a monotherapy or in combination with alternative materials in the context of ARP/ARR and ISD, renders superior clinical and radiographic outcomes when compared with conventional interventions. On the other hand, histomorphometric results are favorably influenced by the adjunctive use of these biologics. PROMs were under‐reported in the included investigations and were minimally influenced by the application of biologics. Given these findings, it is currently not possible to establish recommendations for the clinical use of ABPs, EMD, rhPDGF‐BB, or rhBMP‐2 in ARP/ARR and ISD interventions. Future investigations should focus on conducting well‐designed clinical trials that assess clinical, implant‐related, digital imaging, histologic and patient‐related outcomes in relation to the use of biologics in ARP/ARR and ISD procedures versus a proper control.

## CONFLICTS OF INTEREST

The authors have no conflicts of interest related to this systematic review.

## Supporting information

Supporting Information

Supporting Information

Supporting Information

Supporting Information

Supporting Information
