# Supplementary material for: Efficacy of biologics for alveolar ridge preservation/reconstruction and implant site development: An American Academy of Periodontology best evidence systematic review
Source: J Periodontol. 2022 Oct 24;93(12):1827–47. doi: 10.1002/JPER.22-0069 (PMC10092438; doi:10.1002/JPER.22-0069)
Supplement: Supplementary file 1 — Supporting Information [file JPER-93-1827-s005.docx]

**Supplementary table 1.** Levels of certainty in the body of evidence.

| Level of Certainty in Effect Estimate | | Description |
| --- | --- | --- |
| High | **The body of evidence usually includes consistent results from well-designed, well-conducted studies in representative populations. This conclusion is unlikely to be strongly affected by the results of future studies**.  *This statement is strongly supported by the best available evidence*. | |
| Moderate | **As more information becomes available, the magnitude or direction of the observed effect could change, and this change could be large enough to alter the conclusion.**  *This statement is based on preliminary determination from the current best available evidence, but confidence in the estimate is constrained by one or more factors, such as:*   1. • The limited number or size of studies 2. • Plausible bias that raises some doubt about the results 3. • Inconsistency of findings across individual studies 4. • Imprecision in the summary estimate 5. • Limited applicability due to the populations of interest 6. • Evidence of publication bias, or 7. • Lack of coherence in the chain of evidence. | |
| Low | **More information could allow a reliable estimation of effects on health outcomes.**  *The available evidence is insufficient to support the statement, or the statement is based on extrapolation from the best available evidence. Evidence is insufficient or the reliability of estimated effects is limited by factors such as:*   1. • The limited number or size of studies 2. • Plausible bias that seriously weakens confidence in the results 3. • Inconsistency of findings across individual studies 4. • Imprecision in the summary estimate 5. • Gaps in the chain of evidence 6. • Findings not applicable to the populations of interest 7. • Evidence of publication bias, or 8. • A lack of information on important health outcomes. | |
