# Supplementary material for: Efficacy of biologics for alveolar ridge preservation/reconstruction and implant site development: An American Academy of Periodontology best evidence systematic review
Source: J Periodontol. 2022 Oct 24;93(12):1827–47. doi: 10.1002/JPER.22-0069 (PMC10092438; doi:10.1002/JPER.22-0069)
Supplement: Supplementary file 2 — Supporting Information [file JPER-93-1827-s002.docx]

**Supplementary table 2.** Balancing level of certainty in the benefit estimate (i.e., test over control therapy) with potential for harm.

|  | **Net benefit rating** | | |
| --- | --- | --- | --- |
| **Level of Certainty** | *Clinical benefits outweigh potential harms* | *Modest or uncertain additional clinical benefits outweigh potential harms or benefits balanced with potential harms* | *No clinical benefits or potential harms outweigh benefits* |
| **High** | Strong | In favor | Against |
| **Moderate** | In favor | Weak | Against |
| **Low** | ***Expert opinion for/supports*** | ***Expert opinion for/supports or Expert opinion questions the use*** | ***Expert opinion against*** |
