# Supplementary material for: Efficacy of biologics for alveolar ridge preservation/reconstruction and implant site development: An American Academy of Periodontology best evidence systematic review
Source: J Periodontol. 2022 Oct 24;93(12):1827–47. doi: 10.1002/JPER.22-0069 (PMC10092438; doi:10.1002/JPER.22-0069)
Supplement: Supplementary file 3 — Supporting Information [file JPER-93-1827-s003.docx]

**Supplementary table 3**. Definitions for the strength and direction of clinical recommendation.

| Recommendation strength | Definition |
| --- | --- |
| Strong | Evidence strongly supports the recommendation of this intervention |
| In favor | Evidence favors the recommendation of this intervention |
| Weak | Evidence suggests recommending this intervention after other alternatives have been considered |
| Expert opinion for/supports | Evidence is lacking; the level of certainty is low. Expert opinion guides the recommendation of this intervention. |
| Expert opinion questions the use | Evidence is lacking; the level of certainty is low. Expert opinion questions the recommendation of this intervention. |
| Expert opinion against | Evidence is lacking; the level of certainty is low. Expert opinion suggests not recommending this intervention. |
| Against | Evidence suggests not recommending this intervention (ineffective or harmful). |
