# Supplementary material for: Efficacy of biologics for alveolar ridge preservation/reconstruction and implant site development: An American Academy of Periodontology best evidence systematic review
Source: J Periodontol. 2022 Oct 24;93(12):1827–47. doi: 10.1002/JPER.22-0069 (PMC10092438; doi:10.1002/JPER.22-0069)
Supplement: Supplementary file 4 — Supporting Information [file JPER-93-1827-s001.docx]

**Supplementary table 4.** Excluded articles and reasons for exclusion.

| Reason for exclusion | Reference |
| --- | --- |
| <10 sites | (Khairy et al., 2013, Lee and Jeong, 2020, Nevins et al., 2011, Nevins et al., 2014, Bosshardt et al., 2014, Choukroun et al., 2006, Comert Kilic et al., 2017, Gassling et al., 2013, Moussa et al., 2016, Zhang et al., 2012, Hauser et al., 2013, Anitua et al., 2015, Alkan et al., 2013, Geurs et al., 2014, Olgun et al., 2018, Kaarthikeyan et al., 2019, Kutkut et al., 2012, Badr et al., 2016, Raghoebar et al., 2005, Shim et al., 2018) |
| No or inadequate reporting of outcomes of interest | (Aravena et al., 2021, Nisar et al., 2020, Alissa et al., 2010, Ntounis et al., 2015, Thakkar et al., 2016, Taschieri et al., 2018, Hartlev et al., 2021, Lindeboom et al., 2007, Del Fabbro et al., 2015, Torres et al., 2009, Tatullo et al., 2012, Schaaf et al., 2008a, Schaaf et al., 2008b, Lee et al., 2008, Han et al., 2021) |
| No control therapy without biologics | (Taschieri et al., 2016, Amorfini et al., 2014, Ouyyamwongs et al., 2019, Jo et al., 2019) |
| No use of biologics | (Hermund et al., 2012, Meijndert et al., 2008, Meijndert et al., 2007, de Oliveira et al., 2016, Pelegrine et al., 2010, Rickert et al., 2014, Garcia-Denche et al., 2013, Milani et al., 2016) |
| No implant site development intervention | (Alomari and Sultan, 2019) |
| Immediate implant placement | (Khan et al., 2021) |
| No randomized design | (Bolukbasi et al., 2015) |
| No direct comparison | (Santana et al., 2015) |

**REFERENCES**

Alissa, R., Esposito, M., Horner, K. & Oliver, R. (2010) The influence of platelet-rich plasma on the healing of extraction sockets: an explorative randomised clinical trial. *Eur J Oral Implantol* **3,** 121-134.

Alkan, E. A., Parlar, A., Yildirim, B. & Senguven, B. (2013) Histological comparison of healing following tooth extraction with ridge preservation using enamel matrix derivatives versus Bio-Oss Collagen: a pilot study. *Int J Oral Maxillofac Surg* **42,** 1522-1528. doi:10.1016/j.ijom.2013.06.002.

Alomari, E. B. & Sultan, K. (2019) Efficacy of injectable platelet-rich plasma in reducing alveolar bone resorption following rapid maxillary expansion: A cone-beam computed tomography assessment in a randomized split-mouth controlled trial. *Angle Orthod* **89,** 705-712. doi:10.2319/091018-661.1.

Amorfini, L., Migliorati, M., Signori, A., Silvestrini-Biavati, A. & Benedicenti, S. (2014) Block allograft technique versus standard guided bone regeneration: a randomized clinical trial. *Clin Implant Dent Relat Res* **16,** 655-667. doi:10.1111/cid.12040.

Anitua, E., Murias-Freijo, A., Alkhraisat, M. H. & Orive, G. (2015) Clinical, radiographical, and histological outcomes of plasma rich in growth factors in extraction socket: a randomized controlled clinical trial. *Clin Oral Investig* **19,** 589-600. doi:10.1007/s00784-014-1278-2.

Aravena, P. C., Sandoval, S. P., Pizarro, F. E., Simpson, M. I., Castro-Adams, N., Serandour, G. & Rosas, C. (2021) Leukocyte and Platelet-Rich Fibrin Have Same Effect as Blood Clot in the 3-Dimensional Alveolar Ridge Preservation. A Split-Mouth Randomized Clinical Trial. *J Oral Maxillofac Surg* **79,** 575-584. doi:10.1016/j.joms.2020.10.006.

Badr, M., Oliver, R., Pemberton, P. & Coulthard, P. (2016) Platelet-Rich Plasma in Grafted Maxillae: Growth Factor Quantification and Dynamic Histomorphometric Evaluation. *Implant Dent* **25,** 492-498. doi:10.1097/ID.0000000000000410.

Bolukbasi, N., Ersanli, S., Keklikoglu, N., Basegmez, C. & Ozdemir, T. (2015) Sinus Augmentation With Platelet-Rich Fibrin in Combination With Bovine Bone Graft Versus Bovine Bone Graft in Combination With Collagen Membrane. *J Oral Implantol* **41,** 586-595. doi:10.1563/AAID-JOI-D-13-00129.

Bosshardt, D. D., Bornstein, M. M., Carrel, J. P., Buser, D. & Bernard, J. P. (2014) Maxillary sinus grafting with a synthetic, nanocrystalline hydroxyapatite-silica gel in humans: histologic and histomorphometric results. *Int J Periodontics Restorative Dent* **34,** 259-267. doi:10.11607/prd.1419.

Choukroun, J., Diss, A., Simonpieri, A., Girard, M. O., Schoeffler, C., Dohan, S. L., Dohan, A. J., Mouhyi, J. & Dohan, D. M. (2006) Platelet-rich fibrin (PRF): a second-generation platelet concentrate. Part V: histologic evaluations of PRF effects on bone allograft maturation in sinus lift. *Oral Surg Oral Med Oral Pathol Oral Radiol Endod* **101,** 299-303. doi:10.1016/j.tripleo.2005.07.012.

Comert Kilic, S., Gungormus, M. & Parlak, S. N. (2017) Histologic and histomorphometric assessment of sinus-floor augmentation with beta-tricalcium phosphate alone or in combination with pure-platelet-rich plasma or platelet-rich fibrin: A randomized clinical trial. *Clin Implant Dent Relat Res* **19,** 959-967. doi:10.1111/cid.12522.

de Oliveira, T. A., Aloise, A. C., Orosz, J. E., de Mello, E. O. R., de Carvalho, P. & Pelegrine, A. A. (2016) Double Centrifugation Versus Single Centrifugation of Bone Marrow Aspirate Concentrate in Sinus Floor Elevation: A Pilot Study. *Int J Oral Maxillofac Implants* **31,** 216-222. doi:10.11607/jomi.4170.

Del Fabbro, M., Corbella, S., Ceresoli, V., Ceci, C. & Taschieri, S. (2015) Plasma Rich in Growth Factors Improves Patients' Postoperative Quality of Life in Maxillary Sinus Floor Augmentation: Preliminary Results of a Randomized Clinical Study. *Clin Implant Dent Relat Res* **17,** 708-716. doi:10.1111/cid.12171.

Garcia-Denche, J. T., Wu, X., Martinez, P. P., Eimar, H., Ikbal, D. J., Hernandez, G., Lopez-Cabarcos, E., Fernandez-Tresguerres, I. & Tamimi, F. (2013) Membranes over the lateral window in sinus augmentation procedures: a two-arm and split-mouth randomized clinical trials. *J Clin Periodontol* **40,** 1043-1051. doi:10.1111/jcpe.12153.

Gassling, V., Purcz, N., Braesen, J. H., Will, M., Gierloff, M., Behrens, E., Acil, Y. & Wiltfang, J. (2013) Comparison of two different absorbable membranes for the coverage of lateral osteotomy sites in maxillary sinus augmentation: a preliminary study. *J Craniomaxillofac Surg* **41,** 76-82. doi:10.1016/j.jcms.2012.10.015.

Geurs, N., Ntounis, A., Vassilopoulos, P., Van der Velden, U., Loos, B. G. & Reddy, M. (2014) Using growth factors in human extraction sockets: a histologic and histomorphometric evaluation of short-term healing. *Int J Oral Maxillofac Implants* **29,** 485-496. doi:10.11607/jomi.3408.

Han, J. J., Chang, A. R., Ahn, J., Jung, S., Hong, J., Oh, H. K. & Hwang, S. J. (2021) Efficacy and safety of rhBMP/beta-TCP in alveolar ridge preservation: a multicenter, randomized, open-label, comparative, investigator-blinded clinical trial. *Maxillofac Plast Reconstr Surg* **43,** 42. doi:10.1186/s40902-021-00328-0.

Hartlev, J., Norholt, S. E., Schou, S. & Isidor, F. (2021) Pain after mandibular ramus block harvesting and lateral ridge augmentation with and without involvement of platelet-rich fibrin: a randomized controlled trial. *Int J Oral Maxillofac Surg* **50,** 384-390. doi:10.1016/j.ijom.2020.07.009.

Hauser, F., Gaydarov, N., Badoud, I., Vazquez, L., Bernard, J. P. & Ammann, P. (2013) Clinical and histological evaluation of postextraction platelet-rich fibrin socket filling: a prospective randomized controlled study. *Implant Dent* **22,** 295-303. doi:10.1097/ID.0b013e3182906eb3.

Hermund, N. U., Stavropoulos, A., Donatsky, O., Nielsen, H., Clausen, C., Reibel, J., Pakkenberg, B. & Holmstrup, P. (2012) Reimplantation of cultivated human bone cells from the posterior maxilla for sinus floor augmentation. Histological results from a randomized controlled clinical trial. *Clin Oral Implants Res* **23,** 1031-1037. doi:10.1111/j.1600-0501.2011.02251.x.

Jo, D. W., Cho, Y. D., Seol, Y. J., Lee, Y. M., Lee, H. J. & Kim, Y. K. (2019) A randomized controlled clinical trial evaluating efficacy and adverse events of different types of recombinant human bone morphogenetic protein-2 delivery systems for alveolar ridge preservation. *Clin Oral Implants Res* **30,** 396-409. doi:10.1111/clr.13423.

Kaarthikeyan, G., Jayakumar, N. D. & Sivakumar, D. (2019) Comparative Evaluation of Bone Formation between PRF and Blood Clot Alone as the Sole Sinus-Filling Material in Maxillary Sinus Augmentation with the Implant as a Tent Pole: A Randomized Split-Mouth Study. *J Long Term Eff Med Implants* **29,** 105-111. doi:10.1615/JLongTermEffMedImplants.2019031387.

Khairy, N. M., Shendy, E. E., Askar, N. A. & El-Rouby, D. H. (2013) Effect of platelet rich plasma on bone regeneration in maxillary sinus augmentation (randomized clinical trial). *Int J Oral Maxillofac Surg* **42,** 249-255. doi:10.1016/j.ijom.2012.09.009.

Khan, A. S., Zaheer, N., Zaigham, A. M., Shahbaz, M., Zaheer, U. & Alam, M. K. (2021) Effect of Platelet-Rich Plasma on Bone Healing in Immediate Implants Analyzed by Cone Beam Computerized Tomography: A Randomized Controlled Trial. *Biomed Res Int* **2021,** 6685991. doi:10.1155/2021/6685991.

Kutkut, A., Andreana, S., Kim, H. L. & Monaco, E., Jr. (2012) Extraction socket preservation graft before implant placement with calcium sulfate hemihydrate and platelet-rich plasma: a clinical and histomorphometric study in humans. *J Periodontol* **83,** 401-409. doi:10.1902/jop.2011.110237.

Lee, C. Y., Rohrer, M. D. & Prasad, H. S. (2008) Immediate loading of the grafted maxillary sinus using platelet rich plasma and autogenous bone: a preliminary study with histologic and histomorphometric analysis. *Implant Dent* **17,** 59-73. doi:10.1097/ID.0b013e318166ce3c.

Lee, J. H. & Jeong, S. N. (2020) Effect of enamel matrix derivative on alveolar ridge preservation in the posterior maxilla: A randomized controlled clinical trial. *Clin Implant Dent Relat Res* **22,** 622-630. doi:10.1111/cid.12940.

Lindeboom, J. A., Mathura, K. R., Aartman, I. H., Kroon, F. H., Milstein, D. M. & Ince, C. (2007) Influence of the application of platelet-enriched plasma in oral mucosal wound healing. *Clin Oral Implants Res* **18,** 133-139. doi:10.1111/j.1600-0501.2006.01288.x.

Meijndert, L., Meijer, H. J., Stellingsma, K., Stegenga, B. & Raghoebar, G. M. (2007) Evaluation of aesthetics of implant-supported single-tooth replacements using different bone augmentation procedures: a prospective randomized clinical study. *Clin Oral Implants Res* **18,** 715-719. doi:10.1111/j.1600-0501.2007.01415.x.

Meijndert, L., Raghoebar, G. M., Meijer, H. J. & Vissink, A. (2008) Clinical and radiographic characteristics of single-tooth replacements preceded by local ridge augmentation: a prospective randomized clinical trial. *Clin Oral Implants Res* **19,** 1295-1303. doi:10.1111/j.1600-0501.2008.01523.x.

Milani, S., Dal Pozzo, L., Rasperini, G., Sforza, C. & Dellavia, C. (2016) Deproteinized bovine bone remodeling pattern in alveolar socket: a clinical immunohistological evaluation. *Clin Oral Implants Res* **27,** 295-302. doi:10.1111/clr.12535.

Moussa, M., El-Dahab, O. A. & El Nahass, H. (2016) Anterior Maxilla Augmentation Using Palatal Bone Block with Platelet-Rich Fibrin: A Controlled Trial. *Int J Oral Maxillofac Implants* **31,** 708-715. doi:10.11607/jomi.3926.

Nevins, M. L., Camelo, M., Schupbach, P., Nevins, M., Kim, S. W. & Kim, D. M. (2011) Human buccal plate extraction socket regeneration with recombinant human platelet-derived growth factor BB or enamel matrix derivative. *Int J Periodontics Restorative Dent* **31,** 481-492.

Nevins, M. L., Reynolds, M. A., Camelo, M., Schupbach, P., Kim, D. M. & Nevins, M. (2014) Recombinant human platelet-derived growth factor BB for reconstruction of human large extraction site defects. *Int J Periodontics Restorative Dent* **34,** 157-163. doi:10.11607/prd.1743.

Nisar, N., Nilesh, K., Parkar, M. I. & Punde, P. (2020) Extraction socket preservation using a collagen plug combined with platelet-rich plasma (PRP): A comparative clinico-radiographic study. *J Dent Res Dent Clin Dent Prospects* **14,** 139-145. doi:10.34172/joddd.2020.028.

Ntounis, A., Geurs, N., Vassilopoulos, P. & Reddy, M. (2015) Clinical assessment of bone quality of human extraction sockets after conversion with growth factors. *Int J Oral Maxillofac Implants* **30,** 196-201. doi:10.11607/jomi.3518.

Olgun, E., Ozkan, S. Y., Atmaca, H. T., Yalim, M. & Hendek, M. K. (2018) Comparison of the clinical, radiographic, and histological effects of titanium-prepared platelet rich fibrin to allograft materials in sinus-lifting procedures. *J Investig Clin Dent* **9,** e12347. doi:10.1111/jicd.12347.

Ouyyamwongs, W., Leepong, N. & Suttapreyasri, S. (2019) Alveolar Ridge Preservation Using Autologous Demineralized Tooth Matrix and Platelet-Rich Fibrin Versus Platelet-Rich Fibrin Alone: A Split-Mouth Randomized Controlled Clinical Trial. *Implant Dent* **28,** 455-462. doi:10.1097/ID.0000000000000918.

Pelegrine, A. A., da Costa, C. E., Correa, M. E. & Marques, J. F., Jr. (2010) Clinical and histomorphometric evaluation of extraction sockets treated with an autologous bone marrow graft. *Clin Oral Implants Res* **21,** 535-542. doi:10.1111/j.1600-0501.2009.01891.x.

Raghoebar, G. M., Schortinghuis, J., Liem, R. S., Ruben, J. L., van der Wal, J. E. & Vissink, A. (2005) Does platelet-rich plasma promote remodeling of autologous bone grafts used for augmentation of the maxillary sinus floor? *Clin Oral Implants Res* **16,** 349-356. doi:10.1111/j.1600-0501.2005.01115.x.

Rickert, D., Vissink, A., Slot, W. J., Sauerbier, S., Meijer, H. J. & Raghoebar, G. M. (2014) Maxillary sinus floor elevation surgery with BioOss(R) mixed with a bone marrow concentrate or autogenous bone: test of principle on implant survival and clinical performance. *Int J Oral Maxillofac Surg* **43,** 243-247. doi:10.1016/j.ijom.2013.09.006.

Santana, R. B., Santana, C. M. & Dibart, S. (2015) Platelet-Derived Growth Factor-Mediated Guided Bone Regeneration in Immediate Implant Placement in Molar Sites with Buccal Bone Defects. *Int J Periodontics Restorative Dent* **35,** 825-833. doi:10.11607/prd.2330.

Schaaf, H., Streckbein, P., Lendeckel, S., Heidinger, K., Gortz, B., Bein, G., Boedeker, R. H., Schlegel, K. A. & Howaldt, H. P. (2008a) Topical use of platelet-rich plasma to influence bone volume in maxillary augmentation: a prospective randomized trial. *Vox Sang* **94,** 64-69. doi:10.1111/j.1423-0410.2007.00997.x.

Schaaf, H., Streckbein, P., Lendeckel, S., Heidinger, K. S., Rehmann, P., Boedeker, R. H. & Howaldt, H. P. (2008b) Sinus lift augmentation using autogenous bone grafts and platelet-rich plasma: radiographic results. *Oral Surg Oral Med Oral Pathol Oral Radiol Endod* **106,** 673-678. doi:10.1016/j.tripleo.2008.04.004.

Shim, J. Y., Lee, Y., Lim, J. H., Jin, M. U., Lee, J. M., Suh, J. Y. & Kim, Y. G. (2018) Comparative Evaluation of Recombinant Human Bone Morphogenetic Protein-2/Hydroxyapatite and Bovine Bone for New Bone Formation in Alveolar Ridge Preservation. *Implant Dent* **27,** 623-629. doi:10.1097/ID.0000000000000814.

Taschieri, S., Corbella, S., Weinstein, R., Di Giancamillo, A., Mortellaro, C. & Del Fabbro, M. (2016) Maxillary Sinus Floor Elevation Using Platelet-Rich Plasma Combined With Either Biphasic Calcium Phosphate or Deproteinized Bovine Bone. *J Craniofac Surg* **27,** 702-707. doi:10.1097/SCS.0000000000002522.

Taschieri, S., Lolato, A., Testori, T., Francetti, L. & Del Fabbro, M. (2018) Short dental implants as compared to maxillary sinus augmentation procedure for the rehabilitation of edentulous posterior maxilla: Three-year results of a randomized clinical study. *Clin Implant Dent Relat Res* **20,** 9-20. doi:10.1111/cid.12563.

Tatullo, M., Marrelli, M., Cassetta, M., Pacifici, A., Stefanelli, L. V., Scacco, S., Dipalma, G., Pacifici, L. & Inchingolo, F. (2012) Platelet Rich Fibrin (P.R.F.) in reconstructive surgery of atrophied maxillary bones: clinical and histological evaluations. *Int J Med Sci* **9,** 872-880. doi:10.7150/ijms.5119.

Thakkar, D. J., Deshpande, N. C., Dave, D. H. & Narayankar, S. D. (2016) A comparative evaluation of extraction socket preservation with demineralized freeze-dried bone allograft alone and along with platelet-rich fibrin: A clinical and radiographic study. *Contemp Clin Dent* **7,** 371-376. doi:10.4103/0976-237X.188567.

Torres, J., Tamimi, F., Martinez, P. P., Alkhraisat, M. H., Linares, R., Hernandez, G., Torres-Macho, J. & Lopez-Cabarcos, E. (2009) Effect of platelet-rich plasma on sinus lifting: a randomized-controlled clinical trial. *J Clin Periodontol* **36,** 677-687. doi:10.1111/j.1600-051X.2009.01437.x.

Zhang, Y., Tangl, S., Huber, C. D., Lin, Y., Qiu, L. & Rausch-Fan, X. (2012) Effects of Choukroun's platelet-rich fibrin on bone regeneration in combination with deproteinized bovine bone mineral in maxillary sinus augmentation: a histological and histomorphometric study. *J Craniomaxillofac Surg* **40,** 321-328. doi:10.1016/j.jcms.2011.04.020.
