# Supplementary figures and images for: Efficacy of biologics for alveolar ridge preservation/reconstruction and implant site development: An American Academy of Periodontology best evidence systematic review
Source: J Periodontol. 2022 Oct 24;93(12):1827–47. doi: 10.1002/JPER.22-0069 (PMC10092438; doi:10.1002/JPER.22-0069)

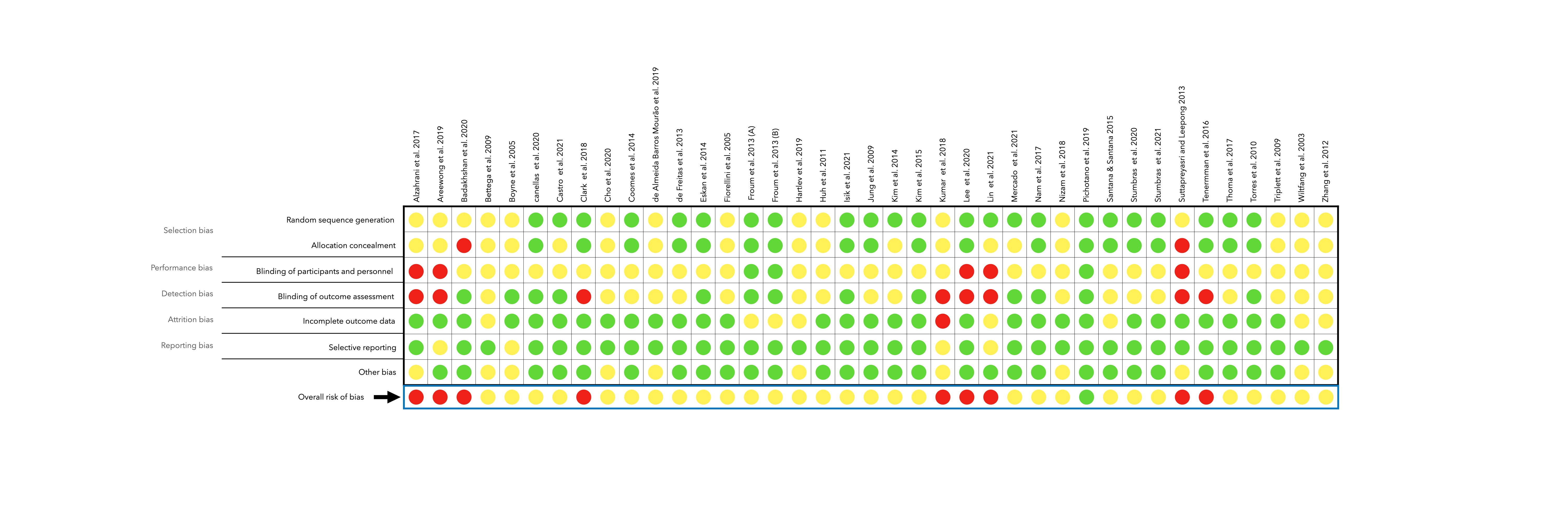

Supplement: Supplementary file 5 — Supporting Information [file JPER-93-1827-s004.jpg]
